# Dopamine and glucose, obesity, and reward deficiency syndrome

**DOI:** 10.3389/fpsyg.2014.00919

**Published:** 2014-09-17

**Authors:** Kenneth Blum, Panayotis K. Thanos, Mark S. Gold

**Affiliations:** ^1^Department of Psychiatry, McKnight Brain Institute, College of Medicine, University of Florida, GainesvilleFL, USA; ^2^Department of Addiction Research & Therapy, Malibu Beach Recovery CenterMalibu Beach, CA, USA; ^3^Behavior Neuropharmacology and Neuroimaging Lab, Department of Psychology, State University of New YorkStony Brook, NY, USA

**Keywords:** obesity, glucose craving, dopamine release, glucoprivation, neurogentics, reward deficiency syndrome

## Abstract

Obesity as a result of overeating as well as a number of well described eating disorders has been accurately considered to be a world-wide epidemic. Recently a number of theories backed by a plethora of scientifically sound neurochemical and genetic studies provide strong evidence that food addiction is similar to psychoactive drug addiction. Our laboratory has published on the concept known as Reward Deficiency Syndrome (RDS) which is a genetic and epigenetic phenomena leading to impairment of the brain reward circuitry resulting in a hypo-dopaminergic function. RDS involves the interactions of powerful neurotransmitters and results in abnormal craving behavior. A number of important facts which could help translate to potential therapeutic targets espoused in this focused review include: (1) consumption of alcohol in large quantities or carbohydrates binging stimulates the brain’s production of and utilization of dopamine; (2) in the meso-limbic system the enkephalinergic neurons are in close proximity, to glucose receptors; (3) highly concentrated glucose activates the calcium channel to stimulate dopamine release from P12 cells; (4) a significant correlation between blood glucose and cerebrospinal fluid concentrations of homovanillic acid the dopamine metabolite; (5) 2-deoxyglucose (2DG), the glucose analog, in pharmacological doses is associated with enhanced dopamine turnover and causes acute glucoprivation. Evidence from animal studies and fMRI in humans support the hypothesis that multiple, but similar brain circuits are disrupted in obesity and drug dependence and for the most part, implicate the involvement of DA-modulated reward circuits in pathologic eating behaviors. Based on a consensus of neuroscience research treatment of both glucose and drug like cocaine, opiates should incorporate dopamine agonist therapy in contrast to current theories and practices that utilizes dopamine antagonistic therapy. Considering that up until now clinical utilization of powerful dopamine D2 agonists have failed due to chronic down regulation of D2 receptors newer targets based on novel less powerful D2 agonists that up-regulate D2 receptors seems prudent. We encourage new strategies targeted at improving DA function in the treatment and prevention of obesity a subtype of reward deficiency.

*Reward Deficiency Syndrome* (RDS; Blum et al., 1996) caused by a Brain Reward Cascade dysfunction is linked to polymorphisms in the Dopaminergic system that cause hypo-dopaminergic function and result in abnormal craving behavior (Zhu and Shih, 1997). Dopamine, a very powerful neurotransmitter, controls feelings of well-being. The complex interactions of powerful neurotransmitters like serotonin, enkephalins, and GABA that ultimately regulates dopaminergic activation of the Reward Center of the brain has been characterized by Blum as “The Brain Reward Cascade” (Blum and Kozlowski, 1990; Blum et al., 2000).

While, for example, high levels of enkephalins are associated with pain suppression and low serotonin levels with depression, an individual with the *Taq1 A1* allele of the Dopamine Receptor Gene (*DRD2*), lacks enough dopamine receptor sites to release the normal amount of dopamine into the Reward Center of the brain and dopamine function is reduced (Noble et al., 1991; Delis et al., 2013). Humans possessing the A1 variant crave and seek substances and behaviors known to cause dopamine release preferentially at the nucleus accumbens (NAc) in the meso-limbic system (Stice and Dagher, 2010). They may become serious cocaine abusers or have unhealthy appetites, which lead to, eating disorders like obesity, overeating or at the other extreme, anorexia nervosa (AN), they also suffer from high levels of stress over an extended period of time. To activate their dopaminergic pathways, a self-healing process to offset their low D2 receptors, individuals are driven to engage in activities which will increase brain dopamine function (Noble et al., 1991, 1993; Delis et al., 2013). The consumption of alcohol in large quantities or carbohydrate binging stimulates the brain’s production of and utilization of dopamine (Blum et al., 1996, 2000). So too does the intake of crack cocaine, cocaine, opioids, and the abuse of nicotine. Aggressive behavior has also been associated with this genetic abnormality which also stimulates the brain’s use of Dopamine (Blum et al., 2000).

Reward deficiency syndrome manifests as a form of sensory deprivation the pleasure or reward mechanisms and can be relatively severe or mild, a consequence of an individual’s neurochemical inability to derive pleasure from ordinary, everyday activities. The A1 variant of the *DRD2* gene generates an alteration in the reward pathways and has been associated in neurogenetic research with a spectrum of addictive, compulsive and impulsive behaviors. The RDS concept unites these disorders and may help to explain how simple genetic anomalies can give rise to complex aberrant behavior (Noble et al., 1991, 1993; Blum et al., 1996, 2000; Delis et al., 2013).

One notable study from Thanos et al. (2001) provides support for the role of the *DRD2* gene in alcohol intake in rats. Utilizing a cDNA construct, a precursor of the *DRD2* gene, was implanted into the NAc of rats. After 4 days of treatment, dopamine D2 receptors increased above pretreatment levels by 150% and alcohol drinking was halved. After 8 days in total, D2 receptor densities and alcohol drinking returned to pretreatment levels. Second injections of the same construct 24 days later, similarly increased DRD2 density, this time, with a twofold decrease in drinking (Thanos et al., 2001). This phenomenon had also been observed for cocaine dependence (Thanos et al., 2008b). In another study the same group (Wang et al., 2001) using positron-emission tomography (PET) scanning techniques, reported low D2 receptor density in the obese subjects compared to non-obese controls. The D2 receptor paucity also correlated with high body mass index (BMI).

In another study, Hamdi et al. (1992) found that dopamine D2 receptor availability in the striatum was significantly lower in obese Zucker rats than in lean non-Zucker controls. Moreover, others have shown the availability of the dopamine striatal dopamine transporter was negatively correlated with BMI in healthy volunteers (Chen et al., 2008), suggesting that the dopamine system regulates BMI. Thus, in obese subjects dopamine deficiency may promote compensatory pathological eating to activate reward circuits. Strategies with the goal of improving dopamine function may be of benefit in treating obese individuals (Curtis and Davis, 2014).

To understand the important relationship between dopamine and glucose, it is beneficial to realize that, in the meso-limbic system the enkephalinergic neurons are in close proximity, to glucose receptors. There are other important connections in the substatia nigra (SN), tuberoinfundibular neurons, globus pallidus, and other brain regions (Haltia et al., 2007).

It is well known that glucose by the actions of an ATP-sensitive potassium channel modulates GABA terminal transmitter release and SN dopamine neuronal activity. In a study, Levin et al. (2001) placed microdialysis probes into both the SN and striatum of male rats to assess the effect of altered SN glucose levels on striatal dopamine release. Striatal DA eﬄux transiently increased by 50% during 50 mM glucose infusion, returning to baseline after 60 min. Moreover, when GABA (A) antagonist bicuculline was added the eﬄux increased by a further 30%. Furthermore, nigral bicuculline alone raised striatal dopamine eﬄux by 31% above basal glucose levels supporting the well-known tonic GABA inhibitory input to the DA neurons. Thus changing SN glucose levels effects striatal dopamine release. Levin and associates suggest that this response may reflect the known effect of glucose on GABA axon terminals in the SN and SN Dopamine neurons, via K(ATP) channel activity and could be the mechanism by which glucose modulates the motor activity involved in food intake (Levin, 2000). Koshimura et al. (2003) found that long – term incubation with a high concentration of glucose increased the capacity of calcium uptake to enhance depolarization-induced dopamine release from Pheochromocytoma 12 (P12) cells. Taken together, these data suggest that highly concentrated glucose activated the calcium channel to stimulate dopamine release from P12 cells.

Bello et al. (2003) found that the rat dopamine transporter was up-regulated in the ventral tegmental area and the NAc of the brain when feeding was restricted with scheduled sucrose access. Moreover, Lee et al. (1988) found that dopamine can lower glucose uptake into rat white adipocytes that do not have dopaminergic receptors, by activating B3 adrenoreceptors. Glucose utilization in the direct and the indirect pathways, of the rat basal ganglia is affected by injection of intrastriatal D1 and D2 dopamine agonists (Conti et al., 2001). Depending on dosage and time after treatment fat intake in rats can be altered by dopamine receptor antagonism. In this regard, both D1 and D2 receptor co-activation significantly increased protein mass while reducing body fat, body weight, food consumption and serum concentrations of triglycerides, free fatty acid glucose, and insulin (Cincotta et al., 1997). Blood glucose studies found a significant correlation between blood glucose concentrations and cerebrospinal fluid concentrations of homovanillic acid the dopamine metabolite (Umhau et al., 2003).

The RDS hypothesis (Blum et al., 1996), embraces the concept that a genetic commonality exists between dopamine activating substances like alcohol, the opiates and even glucose. Evidence now exists that excessive sugar intake, if intermittent, can induce endogenous opioid dependence. In rats, following repeated intake of excessive sugar an opioid antagonist induced neurochemical and behavioral signs of opioid withdrawal. The indices of dopamine/acetylcholine (Ach) imbalance and anxiety were similar in quality to withdrawal from nicotine or morphine, suggesting that the rats had become sugar-dependent (Colantuoni et al., 2001).

In terms of understanding the brain reward cascade there is evidence that serotonergic activation may also influence dopamine D2 receptor function. This is of interest when we consider the so-called “sweet tooth” which has predominantly been associated with serotonin. The work by Kogan et al. (2002) confirmed that DR4004, a putative 5-HT7 receptor antagonist, has functionally activated the dopamine D2 receptor. Neuroanatomical data suggest that there may be an interactive role between NAc Ach and dopamine. There is evidence that NAc Ach is related to natural consummatory behavior, like feeding, as well as, the neural processes that elicit reward from psychostimulants. In this regard, Hajnal et al. (2000) found that cholinergic interneurons in the accumbens have a role in body weight regulation and metabolism. In this context, both stress and dopamine play an important part in the Ach response.

It is well known that in preclinical studies 2-deoxyglucose (2DG), the glucose analog, in pharmacological doses is associated with enhanced dopamine turnover and causes acute glucoprivation. In fact, indications from lines of evidence are that dopamine release is associated with a variety of metabolic stressors that include acute glucose deprivation. Adler et al. (2000) using PET found that synaptic dopamine concentrations were enhanced by 2DG administration. In healthy volunteers, the administration of 2DG was associated with significant striatal dopamine release. These data are important because it further ties glucose levels to dopaminergic activity. Moreover, there is a relationship between dopamine release and insulin levels in the tuberoinfundibular neurons. This insulin effect is dependent on the CA++ ions, protein kinase C Na (+) – H + exchange system. Additionally, when glucose in the brain is lowered and leads to global cerebral transient ischemia, monamine release of dopamine, especially, is inhibited. In this regard, Trugman and James (1993) showed that D1 antagonists lowered glucose utilization by 24–28%; in the globus pallidus, entopeduncular nucleus, subthalamic nucleus, SN, and in the motor cortex, suggesting that by stimulation of the D1 receptor, endogenous dopamine makes a contribution in these regions to basal metabolism. In contrast with these results both D1 and D2 agonists increase glucose utilization suggesting that stimulation of D1 and D2 receptors is tied to feeding behavior. Thus, the importance of the D1 and D2 functional linkage in the brain is established by this metabolic evidence, which relates to overeating (hyperphagia).

That dopamine induces hyperglycemia in both animals and man is well known. The direct effects of dopamine on the release of glucose from primary cultured rat hepatocytes were studied in Japan by Shiroyama et al. (1998). The authors investigated the effect of dopamine on glucose release through the gluconeogenic and/or glycogenolytic pathways and found the main adrenergic receptor type beta 2, involved in glucose release. The hypothesis is that increasing the release of glucose from tissue would reduce cravings for carbohydrates and glucose. In this regard Shiroyama et al. (1998) supported this notion. Glycogen-rich and gluconeogenic-depleted hepatocytes were prepared in order to study glycogenolytic and gluconeogenic-depleted glucose release, respectively. Dopamine was shown to cause the release of glucose and the beta blocker propranolol was shown to inhibit this release. The authors conclude that mediated by beta adrenergic receptors dopamine has a direct effect on hepatocytes of increasing glucose release in the glycogenolytic and gluconeogenic pathways.

Freeman et al. (2001) studied the effect of glucose on changes induced in dopamine neuronal activity by anti-psychotic drugs and suggested antipsychotic drug-induced changes in midbrain dopaminergic neuron population activity may be influenced by caloric intake. Glucose did, in fact, reduced significantly the number of A9 and A10 dopaminergic cells that were spontaneously active per track in control rats, but attenuated significantly the chronic haloperidol- and clozapine-induced reductions in dopaminergic cells per track.

Certainly, the compulsion and the loss of control observed in the drug taking behaviors of drug-addicted subjects is similar to overeating by obese individuals. Although not well understood the mechanisms of these behaviors were studied by Michaelides et al. (2012) utilizing PET in drug-addicted subjects. Reductions in striatal DA D2 receptors were documented. In pathologically obese subjects, the same researchers found striatal DA D2 receptors reductions similar to those found in drug-addicted subjects. Moreover, DA D2 receptor levels were inversely related to the BMI of the obese subjects. Michaelides et al. (2012) postulated that decreased DA D2 receptors levels predisposed subjects to search for reinforcers; drug of choice in the case of drug-addicted subjects and food in the case of the obese subjects to compensate temporarily for a decreased sensitivity of reward circuits regulated by the activity of DA D2 receptors.

Discovery of strategies for the treatment of obesity will be assisted by better understanding of the mechanisms involved in food intake. Stice et al. (2010, 2012, 2013) and Stice and Yokum (2013) researched these mechanisms and found that carriers of the DRD2 A1 allele and other reward gene polymorphisms have a blunted response to palatable food reward and carriers of D2 and D4 polymorphisms also gained weight in a 1-year follow-up. Recent studies from Stice et al. (2013) showed an elevated brain reward response to money cues in adolescents with a parental substance use disorder, and they suggested support for the reward surfeit model rather than the reward deficit model and as such it is different from prediction of obesity. Stice et al. (2013) may not have considered the role of supersensitive D2 high receptors as suggested by Seeman and Seeman (2014). This is a very complex mechanism involving epigenetic effects in cases of substance use especially in parental substance abuse. The well-known high risk for relapse in carriers of the *DRD2* AI allele (Dahlgren et al., 2011) could be in part due to proposed dopamine receptor supersensitivity (Blum et al., 2009). Furthermore, decreased reward and negative eating behaviors in obesity are accompanied by diminished dopaminergic neurotransmission. Bariatric surgery the most successful therapy for obesity rapidly reduces hunger and improves satiety, the mechanisms are unknown and little is known about dopaminergic activity following this surgical procedure. Dunn et al. (2010) has hypothesized that after Vertical Sleeve Gastrectomy (VSG) or Roux-en-Y-Gastric Bypass (RYGB) surgery dopaminergic neurotransmission would be affected, influence eating behaviors and would contribute to the positive outcomes from bariatric surgery. The results of their study reported an expected body weight decreased and a decrease in DA D2 receptor availability after surgery. These changes were accompanied by significant decreases in plasma insulin of (62%) and plasma of leptin (41%), regional decreases in DA D2 receptors (mean ± SEM) were putamen 9 ± 4%, ventral striatum 8 ± 4%, caudate 10 ± 3%, amygdala 9 ± 3%, hypothalamus 9 ± 3%, substantia nigra 10 ± 2%, and medial thalamus 8 ± 2%. Volkow and associates (Dunn et al., 2010) point out that decreased DA D2 receptor availability following VSG and RYGB is most likely reflected in increased levels of extracellular dopamine. Although better dopaminergic neurotransmission may improve eating behavior with improved satiety and reduced hunger after bariatric procedures, in the longer term a decrease in brain D2/D3 receptor availability may enhance addiction liability and addiction transfer or even cross tolerance. The finding of decreased D2/D3 availability may explain in part the increased risk of drug seeking behavior reported following bariatric surgery. Our hypothesis that the real culprit in obesity may be RDS is supported by this finding (Blum et al., 2011a,b). Increased alcohol intake following bypass surgery was reported by Hajnal et al. (2012) and a reduced reward-related (e.g., striatal) neural activation has been observed following bariatric surgery. Studies by Ochner et al. (2012) reveal that post-operatively reduced mesolimbic responsively was associated with reductions in wanting, high-versus low-calorie foods but not in liking for high caloric foods. These findings support the hypothesized delineation between wanting and liking; the idea that wanting, but not liking is a dopaminergic reward pathway process (Blum et al., 2012a).

Interestingly, in animal models a predisposition in offspring to food addiction was caused by feeding rat mothers fatty, sugary, and salty snacks (junk food) during pregnancy and lactation. Compared to controls rat offspring demonstrated an increase in weight and BMI, their mothers displayed binge eating and junk food overeating behaviors (Ong and Muhlhausler, 2011). These observations may be of relevance to pregnant women with eating disorders and obese women treated with bariatric surgery, in order for them to have healthy children with normal appetites and weight. One must also consider the negative consequences of the hypodopaminergic genetics involved in RDS including obesity.

In support of hypodopaminergic genetics and sugar addiction Avena et al. (2012), found clear evidence that sugar shares the characteristics of addiction neurochemicals, since, like addictive substances, it releases both opioids and dopamine. These authors Avena et al. (2013a,b) classified sugar as addictive, because it follows the typical addiction pathway consisting of binging, withdrawal, craving and cross-sensitization delineated by Blumenthal and Gold (2010) and Blum et al. (2011a,b).

In fact, cross-sensitization was observed in rats showing the movement from to sugar to drugs (Gosnell, 2005). Surprisingly work by Cantin et al. (2010) on a comparative evaluation of the large majority of rats with a history of cocaine addiction, cocaine is valued similarly to the lowest concentrations of sweet water. Additionally, all experiments from the previous 5 years were evaluated. The retrospective analysis revealed that most rats will give up cocaine use in favor of saccharine, the non-drug alternative. A minority, at the heaviest level of past cocaine use <15%, continued to take cocaine and in spite of being hungry, chose cocaine rather than a natural sugar that could relieve their need for calories. Most importantly Cole et al. (1990) suggest that initiation into addiction requires, sensitization and cross tolerance, thus, this model fits for sugar. It is of interest that the withdrawal from sugar induces imbalances in both Ach and dopamine similar to opiate withdrawal. Specifically, Avena et al. (2008) using microdialysis found an increase in extracellular Ach and a decrease in dopamine release, in the NAc shell, in rats undergoing withdrawal from sugar binging. This finding suggest that a state, that involves anxiety, an altered accumbens dopamine and Ach balance is induced by intermittent binging on sucrose and chow followed by fasting. This is similar to withdrawal from opiates following naloxone and may be a feature of some eating disorders.

While there are these similarities between the addictiveness of food and drugs, its validity as a model of obesity has been questioned based on the idea that food is not a psychoactive drug (Sansone and Sansone, 2013). With that said, at the Columbia University Seminar on Appetitive Behavior, the concept of “food Addiction” was one of various proposed causes of the obesity epidemic. This has been vigorously debated in the media (Avena, 2010), as well as in the scientific community (Michaelides et al., 2013). Moreover, the criteria in the Diagnostic & Statistical Manual of Mental Disorders, Fifth Edition (DSM-5) pertaining to substance abuse, has been applied to food addiction in humans based on ever increasing evidence in animal and humans (Gold and Avena, 2013). In terms of sugar being considered a psychoactive substance, clinical accounts from self-identified food addicts describe using food to self-medicate; they eat in order to change a negative mood state (Blum et al., 2013). Behaviors reported by self-identified food addicts conform to the seven DSM-5 criteria for substance use disorders (Campbell et al., 2013). This notion of commonality has been confirmed by studies that show that food craving, in both normal weight and obese patients, activates similar areas of the brain to those indicated in drug seeking (Wise, 2013). Avena (2010) adequately defined *binging, withdrawal, and craving* by presenting evidence from animal models of binge eating of sucrose or glucose, in a review that summarized evidence for “food addiction.” In a *PANTHER* analysis of gene array expression performed on 152 unique genes, resulting in a total of 193 multiple-factor (MF) assignments, sorted into 20 categories (Avena et al., 2010; Blum et al., 2012a,b) found gene clusters expressed significantly differently, in the *ad libitum* sucrose group compared to the sucrose binge eating group. These clusters seem to be convergent with the neurotransmitters involved in the brain reward circuitry like serotonin; endorphins; GABA; dopamine; cannabinoids; ACH and leptin, and specifically in the brain reward cascade (Yarnell et al., 2013) and RDS (Downs et al., 2013).

## CONTROVERSIAL FINDINGS

Since the original finding by Blum et al. (1990) first to associate the *Taq-A1* of the dopamine D2 receptor gene polymorphism and severe alcoholism there have been controversial findings possibly due to poor control screening. One example of poor screening and negative findings relative to the role of dopaminergic gene polymorphisms and reward seeking behavior as well as parenting is observed in the work of Creemers et al. (2011) from a Dutch general population. This problem exists even in the current literature.

We have cautioned against including RDS behaviors in the control group which could lead to spurious results. Since that time there have been no less than 3738 (Pubmed-6-23-14) peer reviewed articles on many peripheral and central nervous system (CNS) behaviors and physiological processes. Understandably addiction or even the broader term RDS involves very complex gene × environment interaction and one cannot expect that a single gene like the DRD2 gene would have a powerful effect by itself, however, albeit many negative findings, there is still a plethora of evidence for the role of the DRD2 gene polymorphisms and a number (small sample of studies represented herein) of addictive and other reward dependent behaviors including: *alcohol dependence* (Pato et al., 1993; Ponce et al., 2003; Munafò et al., 2007; Smith et al., 2008; Pinto et al., 2009; Grzywacz et al., 2012; Wang et al., 2013); *drug dependence* (Li et al., 2004; Xu et al., 2004; Young et al., 2004; Barratt et al., 2006; Li et al., 2006; Hou and Li, 2009; Chen et al., 2011a,b; Al-Eitan et al., 2012; Jacobs et al., 2013; Lee et al., 2013; Ohmoto et al., 2013; Sullivan et al., 2013; Suraj Singh et al., 2013; Vereczkei et al., 2013; Wang et al., 2013; Clarke et al., 2014; Roussotte et al., 2014; Schuck et al., 2014); *mood disorders* (Vaske et al., 2009; Huertas et al., 2010; Zhu et al., 2011; Zou et al., 2012; Hettinger et al., 2012; Jutras-Aswad et al., 2012; Tsuchimine et al., 2012; Whitmer and Gotlib, 2012; Zai et al., 2012; Peciña et al., 2013; Zhang et al., 2014); *rearing behaviors* (Mills-Koonce et al., 2007; Bakermans-Kranenburg and van Ijzendoorn, 2011; Beaver and Belsky, 2012; Masarik et al., 2014); *obesity* (Spangler et al., 2004; Fang et al., 2005; Huang et al., 2005; Epstein et al., 2007; Nisoli et al., 2007; Barnard et al., 2008; Blum et al., 2008; Eny et al., 2009; Epstein et al., 2010; Mathes et al., 2010; Stice et al., 2010; van Strien et al., 2010; Jabłoński, 2011; Anitha et al., 2012; Chen et al., 2012; Winkler et al., 2012; Ariza et al., 2013; Carpenter et al., 2013; Cameron et al., 2013; Hess et al., 2013; Alsiö et al., 2014); *Anorexia Nervosa* (Bergen et al., 2005); *motivation* (Trifilieff et al., 2013); *brain metabolism* (Noble et al., 1997); *ADHD* (Gold et al., 2014), and *pathological gambling* (Gyollai et al., 2014).

It has been argued that the significance of the *Taq 1A* polymorphism is presumed to be related to decreased nucleus accumbens neurotransmission leading to reward deficiency. While human imaging studies have reported lower levels of striatal DA D2 receptors in subjects with the *Taq 1A* polymorphism, the significance is less clear. In subjects with the *Taq 1A* polymorphism 18F 6FDOPA studies have reported significantly increased striatal uptake of 18F 6FDOPA consistent with increased DA synthesis. If there is increased DA synthesis and release, the decreased apparent levels of striatal DA D2 receptors may be due to increased extracellular DA levels. Increased synthesis may be due to a decrease in striatal D2 auto-receptors. While this may be correct the surfeit theory of drug dependence may be incorrect. In fact, surfeit concepts have been also made with regard to escalation of cocaine abuse claiming that the increased abuse is due to increased dopaminergic activity in the accumbens. However, recent clear evidence from Willuhn et al. (2014) dispels this and suggests otherwise that escalation of cocaine abuse is due to low dopaminergic function. In fact utilizing sophisticated analyses they suggested that agonistic not antagonistic intervention would be prudent in terms of treating all addictions.

In terms of BMI and dopaminergic gene polymorphisms and subsequent associations there is controversy especially with regard to the dopamine transporter gene. As stated earlier Chen et al. (2008) reported a significant negative correlation between BMI and striatal DAT1 levels. However, others did not find this association in so called obese healthy subjects (once again not very well screened for RDS behaviors), albeit a larger cohort in the van de Giessen et al. (2013). In addition this non-association was also reported by Thomsen et al. (2013) as well utilizing so called healthy obese subjects. However, there are number of other reports which support the DAT1 negative association with BMI including: Need et al. (2006), Fuemmeler et al. (2008), Wang et al. (2011), Sikora et al. (2013), and Valomon et al. (2014). With these studies in favor of an association of dopaminergic gene polymorphisms and even a study showing that methamphetamine known to block DAT1 reducing fat and carbohydrate intake (Danilovich et al., 2014), there is real controversy concerning the actual role of BMI as a biological marker for obesity relative to percent body fat, as clearly pointed out by Shah and Braverman (2012). This notion is highlighted in a study from Chen et al. (2012) that shows a significant correlation with carriers of the DRD2 *Taq-A1* and higher percent body fat compared to DRD2 *Taq-A2.*

There is some controversy concerning a conclusive statement that sugar addiction may lead to obesity (Hone-Blanchet and Fecteau, 2014). However, the evidence seems to favor a bond between Substance Use Disorders, as clinically categorized in the DSM 5, and food reward (see Brownell, 2012) including an article by Gold and Avena (2013).

In terms of eating disorders there have been a number of reports indicating the potential link between reward gene polymorphisms and binge eating (Davis et al., 2008). The finding that obese carriers of the DRD2 *Taq-A1* allele had higher reward sensitivity (binging behavior) compared to normal weight controls favors the surfeit theory rather than the deficit RDS concept. While this might be true we must caution against these findings because the controls in the study may not reflect a phenotype free of all RDS behaviors which could lead to spurious results. The suggestion of blocking Dopamine activity in the reward circuitry may be of interest in the short term but damaging in the long term as discussed in this article (Blum et al., 2012a). Interestingly, Gearhardt et al. (2011) also found differential neuro-correlates to food scores in healthy young women lean to obese. Food addiction scores correlated with greater activation in the anterior cingulate cortex, medial orbitofrontal cortex, and amygdala in response to anticipated receipt of food. Furthermore, food addiction scores showed greater activation in the dorsolateral prefrontal cortex and the caudate in response to anticipated receipt of food but less activation in the lateral orbitofrontal cortex in response to receipt of food. This particular study portrays the complexity of attempting to dissect brain reward function related to eating behavior.

While there is evidence from Stice’s group that polymorphisms in both dopamine D2 and D4 result in a blunted response to palatable foods and subsequent weight gain (Stice et al., 2008a,b,c, 2010) a paper by Stice et al. (2011) showed in youth that increased striatal dopamine neurotransmission may also be a risk factor for obesity using fMRI. Certainly this supports the surfeit dopamine theory proposed by Robinson and Berridge (2000), and correctly suggests that obesity is a complex disorder and based on both genetics and environment (epigenetics) individuals having increased motivation for food may fall into two categories ( based on gender, age of onset, etc.) either deficit or surfeit in terms of dopaminergic function. More research is required to carefully dissect these differences in the future in terms of “liking and wanting” (Blum et al., 2012a; Willuhn et al., 2014).

We have discussed the potential problems associated with bariatric surgery such as transfer of addiction (Blum et al., 2011a,b) and the work of Dunn et al. (2010) revealing reduced D2R availability (hypodopaminergic state) following bariatric surgery suggestive of increased requirement for self- administered drugs or behaviors linked to dopaminergic activation. Interestingly in five obese subjects Steele et al. (2010) found that pre bariatric surgery the obese subjects had a lower D2 R availability compared to post-surgery levels 6 weeks after surgery whereby it was found that D2R availability increased. This of cause would suggest reduced drug and or addictive behavioral seeking behaviors linked to enhanced dopaminergic function. The question which remains is that the findings by Dunn et al. (2010), was post 7 weeks compared to 6 weeks by Steele et al. (2010) and could this represent a downward trend leading to once again a hypodopaminergic trait. An important question as it relates to our proposed theories regarding transfer of addiction following even longer periods post-bariatric surgery seems prudent.

In addition while we have pointed out that there is evidence for a decreased availability of D2R in obese subjects (Volkow et al., 2009) there is some controversy that argues this is only true for severe obesity (Eisenstein et al., 2013; Kessler et al., 2014). Once again we evoke the concept that using BMI as a factor is not an appropriate phenotype (other RDS behaviors may be a confound variable) and that mild obesity may not indicate the real disorder. This has been underscored by the need for alcoholism severity as the true endophenotype (Blum et al., 1990; Connor et al., 2002). Importantly, Volkow’s group have now published at least 13 papers supporting their original concept of low D2R availability and obesity (Tomasi and Volkow, 2013), however lowered D2R availability was not found with novelty seeking in obesity (Savage et al., 2014).

While additional studies are required to determine the link between dopaminergic function and AN as well as other eating disorders, there are a number of neurogenetic reports. Gervasini et al. (2013) showed a number of interesting associations with dopamine gene polymorphisms including: DRD4 variable number of tandem repeats (VNTR) 7R/7R was significantly associated with greater risk for AN; significant differences in asceticism scores between DAT1 VNTR genotypes; and significant differences in Drive for Thinness and Body Dissatisfaction between DRD4- C-616G genotypes. Moreover, Nisoli et al. (2007) found that independent of obesity, the A1+ allele, both in A1/A1 and A1/A2 genotypes of the DRD2 gene significantly associated with the Drive for thinness and Ineffectiveness. The A1+ allele, both in A1/A1 and A1/A2 genotypes, was not differently distributed among disease groups; on the contrary two EDI subscales (Drive for thinness and Ineffectiveness) resulted in association with A1+ allele without effect of the eating disease or obesity. This finding suggested that the A1+ allele is not simply related to body weight but the A1+ allele is a marker of a genetic psychological trait in humans with high risk to develop pathological eating behavior. In fact other work from National Institute of Alcohol Abuse & Alcoholism (NIAAA) has clearly shown significant linkage disequilibrium between the - 141 Indel and two exon seven SNPs (939Y and 957Y) of the DRD2 gene was observed over a distance of >50 kbp in the AN probands but not in the controls. This further suggests that transmitted variation in D2 dopamine receptor affecting transcription and translation efficiency plays a role in vulnerability to AN.

## KB220 COMPLEX

It is of interest that the complex KB220Z and variants thereof have overcome brain reward circuitry abnormalities, in protracted abstinent psychostimulant abusers observed using qEEG analysis. In fact, KB220Z following only one oral dose of 24 g resulted in an increase in alpha bands with a concomitant increase in low beta bands after 1 h, an effect which usually requires 10–20 biofeedback sessions. This is further supported by preliminary work in China using fMRI showing direct significant activation of dopaminergic pathways compared to placebo during the resting state (Blum et al., 2012b). Thus if KB220Z stimulates dopamine release then it is quite possible that the released dopamine will have an impact on glucose release, which could offset abnormal glucose or even food cravings. We must await further required research to determine the benefits induced by this putative natural D2 agonist especially investigating functional magnetic resonance imaging resting state functional connectivity (rsfMRI) in rodents.

In summary, typically, obesity is associated with abnormal eating behaviors. Brain imaging studies, in both humans and animal models, for the most part, implicate the involvement of DA-modulated reward circuits in pathologic eating behaviors. It is known that food cues increase striatal extracellular DA, providing evidence for the role of DA in the non-hedonic motivational properties of food (Wang et al., 2009). In addition, food cues also increase brain metabolism in the orbitofrontal cortex suggesting the association of this region with enhanced “wanting” of food consumption. Importantly, similar to drug-dependent subjects, striatal DA D2 receptor availability is decreased in obese subjects, which may induce them to seek food (glucose and high fat) as a means to compensate temporarily for under-stimulated (deficient) reward circuits (Thanos et al., 2008a). Reduced DA D2 receptor densities are also associated with reduced responsively in both striatal and prefrontal regions involved in inhibitory control, which may provoke their inability to control food intake and as such weight gain. Interestingly, gastric stimulation especially in obese subjects activates limbic and cortical regions. These same brain regions are activated during drug craving in drug dependent individuals (Cyders et al., 2013). Moreover, obese subjects have enhanced sensitivity to the sensory properties of food. This fact coupled with a reduction of DA D2 receptors places obese subjects at high risk for uncontrollable eating behavior. As noted, in bypass surgery when eating is not an option, there is a transfer of addictive – like behaviors and subsequently drug – seeking may become the new reinforcement (Thanos et al., 2013).

Thus, we submit that results from these on-going investigations indicate that multiple, but similar brain circuits are disrupted in obesity and drug dependence. We encourage new strategies targeted at improving DA function in the treatment and prevention of obesity a subtype of reward deficiency (Avena et al., 2013a,b).

## AUTHOR CONTRIBUTIONS

The authors contributed equally to this manuscript and all provided approval.

## Conflict of Interest Statement

Kenneth Blum is the holder of a number of patents in both the US and foreign countries involved with genetic testing and natural D2 agonistic activity to treat RDS including obesity. He is currently the owner of IGENE, LLC., Synaptamine, Inc. and co-owner of Victory Nutrition, LLC., and RD Solutions LLC. He is a paid consultant from Malibu Beach Recovery Center, Path Foundation NY, USA, RDSolutions, Victory Nutrition, and Chief Scientific Advisor Dominion Diagnostics, LLC. Mark S. Gold is a paid consultant of Malibu Beach Recovery Center. There no other conflicts to report.
